# Emerging Concepts of Mechanisms Controlling Cardiac Tension: Focus on Familial Dilated Cardiomyopathy (DCM) and Sarcomere-Directed Therapies

**DOI:** 10.3390/biomedicines12050999

**Published:** 2024-05-02

**Authors:** R. John Solaro, Paul H. Goldspink, Beata M. Wolska

**Affiliations:** 1 Department of Physiology and Biophysics, Center for Cardiovascular Research, University of Illinois at Chicago, Chicago, IL 60612, USA; pgolds@uic.edu (P.H.G.); bwolska@uic.edu (B.M.W.); 2 Department of Medicine, Section of Cardiology, University of Illinois at Chicago, Chicago, IL 60612, USA

**Keywords:** sarcomere disease genes, TTI, myosin control mechanisms, sarcomere activators, precision medicine

## Abstract

Novel therapies for the treatment of familial dilated cardiomyopathy (DCM) are lacking. Shaping research directions to clinical needs is critical. Triggers for the progression of the disorder commonly occur due to specific gene variants that affect the production of sarcomeric/cytoskeletal proteins. Generally, these variants cause a decrease in tension by the myofilaments, resulting in signaling abnormalities within the micro-environment, which over time result in structural and functional maladaptations, leading to heart failure (HF). Current concepts support the hypothesis that the mutant sarcomere proteins induce a causal depression in the tension-time integral (TTI) of linear preparations of cardiac muscle. However, molecular mechanisms underlying tension generation particularly concerning mutant proteins and their impact on sarcomere molecular signaling are currently controversial. Thus, there is a need for clarification as to how mutant proteins affect sarcomere molecular signaling in the etiology and progression of DCM. A main topic in this controversy is the control of the number of tension-generating myosin heads reacting with the thin filament. One line of investigation proposes that this number is determined by changes in the ratio of myosin heads in a sequestered super-relaxed state (SRX) or in a disordered relaxed state (DRX) poised for force generation upon the Ca^2+^ activation of the thin filament. Contrasting evidence from nanometer–micrometer-scale X-ray diffraction in intact trabeculae indicates that the SRX/DRX states may have a lesser role. Instead, the proposal is that myosin heads are in a basal OFF state in relaxation then transfer to an ON state through a mechano-sensing mechanism induced during early thin filament activation and increasing thick filament strain. Recent evidence about the modulation of these mechanisms by protein phosphorylation has also introduced a need for reconsidering the control of tension. We discuss these mechanisms that lead to different ideas related to how tension is disturbed by levels of mutant sarcomere proteins linked to the expression of gene variants in the complex landscape of DCM. Resolving the various mechanisms and incorporating them into a unified concept is crucial for gaining a comprehensive understanding of DCM. This deeper understanding is not only important for diagnosis and treatment strategies with small molecules, but also for understanding the reciprocal signaling processes that occur between cardiac myocytes and their micro-environment. By unraveling these complexities, we can pave the way for improved therapeutic interventions for managing DCM.

## 1. Introduction

Genetic dilated cardiomyopathy (DCM) Entry-#115200-CARDIOMYOPATHY, DILATED, 1A; CMD1A-OMIM, which accounts for ~40% of the cases of dilated cardiomyopathy, is a complex multi-factorial cardiac disorder lacking a cure and in need of patient-specific therapeutic strategies [1,2,3,4]. The progression of the disorder to heart failure (HF) includes an exacerbating wall thinning, fibrosis, arrythmias, dyspnea, exercise intolerance, and in some cases sudden death. DCM presents as a complex disorder with etiologies that can be either genetic or acquired. Understanding the genetic basis of idiopathic DCM is crucial, as a single gene and the ratio of wild-type and mutant genes can underlie multiple forms of the disease, thereby underscoring the importance of the genomic context in the pathogenesis of disease-associated variants. Genotype–phenotype correlations are being increasingly used for risk assessment and patient management, albeit the prevalence of genetic DCM remains uncertain with estimates ranging widely from 1:250 to 1:2500 in European populations [5]. Although many gene variants are thought to be causative for DCM, recent efforts based on clinical and experimental evidence have narrowed down the number of genes with high evidence supporting their monogenic relationship with idiopathic DCM to 19, including 11 with definite evidence (*BAG3*, *DES*, *FLNC*, *LMNA*, *MYH7*, *PLN*, *RBM20*, *SCN5A*, *TNNC1*, *TNNT2*, *TTN*), 1 with strong evidence (DSP), and 7 with moderate evidence (*ACTC1*, *ACTN2*, *JPH2*, *NEXN*, *TNNI3*, *TPM1*, *VCL*) [6].

A challenge to treatment strategies is the variability of the disease-causing genes, the variability of penetrance, and the variability of the clinical course. Disease genes include variants expressing proteins involved in sarcomere function, in mechano-transduction, in cell and nuclear membrane function, in the cytoskeletal organization of the sarcomere, and in mitochondrial metabolic protein energetic processes [1,7]. Therapies with the objective of maintaining cardiac function in DCM are generally not patient-specific and treat symptoms but not the cause. Treatments include the use of agents such as angiotensin–neprilysin inhibition, Na–glucose co-transport inhibitors, and invasive approaches such as resynchronization therapies [8,9,10]. With a need for a bridge to cardiac transplant, energy-wasting inotropic agents elevating intracellular Ca^2+^ are also employed with the danger of arrythmias and increased mortality [11].

Our focus here is on the disease genes expressing mutant sarcomere proteins that modify tension-generating capability of the sarcomeres triggering disease progression [12]. One rationale for discussing this form of DCM is that in the context of precision medicine, there is a need for deep understanding of the pathological processes, diagnostic approaches, and therapies. A further rationale is that in the case of mutant sarcomere proteins, it appears success is within reach in achieving this understanding and making advances in therapies with small molecules. Although there has been limited success in developing small-molecule sarcomere activators, such as omecamtiv mecarbil (OM) and danicamtiv for use in DCM, the approaches provide evidence that the direct modification of the sarcomere response to Ca^2+^ remains feasible [13,14,15]. On the other hand, there has been success in the clinical use of mavacampten (Camzyos^®^) and aficamten in patients with obstructive hypertrophic cardiomyopathy (HCM) [16,17]. In contrast to the potential use of oral small-molecule therapy in DCM linked to variants of many sarcomere-expressing genes, more complex treatments such as gene therapy and modified gene processing are required for prevalent DCM disease genes such as the following: (i) variants of *TTN*, which express the giant protein titin responsible for resting tension and length-dependent signaling, and (ii) variants of *LMNA* that express lamins that function in nuclear structure control of binding of regulatory elements and chromatin. As detailed in a recent assessment of approaches in gene therapy, the authors concluded that whereas promising techniques are emerging, they are in early development and require ethical and safe use [18]. Thus, although therapies that modify the expression of gene variants linked to DCM are patient-specific, many challenges remain in the use of this approach [2,18].

## 2. Recent Diverse Theories of Control Mechanisms of Tension Development in Cardiac Sarcomeres

An important advance in the understanding of the pathology and treatment strategies in DCM gene variants expressing sarcomere proteins is the identification of a correlation of DCM with a fall in the tension-time integral (TTI) in linear strips of heart muscle [19]. The TTI is the time integral of tension developed in a twitch, for example, in isolated trabeculae at 30 °C and a constant 2.2 µm sarcomere length. The idea that TTI is a critical determinant of adverse signaling in the instigation and the progression of DCM gives rise to the following question: what is the correlation of TTI with muscle function in the beating heart? We think that wall stress is an appropriate variable correlating tension in a linear strip of muscle or cells with the mechanics of the beating heart. As illustrated in the early studies of Sandler and Dodge [20], tension developed by regions of the beating heart is a complex function of chamber pressure and chamber dimensions (size and shape) related to the Law of LaPlace. Early studies by Sandler and Dodge provided a summary of these complexities and a cogent analysis of the determinants of wall stress related explicitly to wall tension [20]. Davis et al. [19] employed computational approaches in the context of various DCM models linked to a variety of gene variants and concluded that the TTI of linear muscle and cardiac myocytes derived from human inducible pluripotent stem cells (hiPSC-CMs) can predict heart thickening in HCM and thinning in DCM. A follow-up study by Powers et al. [12] supported this concept in investigations of TTIs in preparations from a mouse model harboring a DCM-linked tropomyosin mutant (Tm-D230N). However, clouding the understanding of the effects of TTI in triggering DCM is a lack of a unified theory of tension generation and control in the heart. A related and important issue is that the most common causes of DCM are truncation mutants of titin in which studies with skinned muscle preparations report an increased myofilament response to Ca^2+^ [21]. DCM-linked mutations in titin induce truncations responsible for a complex pathology involving adverse post-translational modifications, co-morbidities, and the activation of other maladaptive genes [22]. There are also possible adverse effects of both haploinsufficiency and poison peptides [23]. Recent publications discussed below regarding the generation and control of tension in heart muscle indicate the need to re-evaluate recent ideas presented regarding the relation between TTI, heart growth, and adverse remodeling.

Despite the many years of studies investigating mechanisms of tension generation with each beat of the heart, there is a confluence of new information from integrated molecular/biochemical, cellular, biophysical, and structural studies that demand altered thinking of not only how a DCM point mutation in a sarcomere protein alters the TTI in DCM, but also as to how this mechanical signal is transduced to adverse remodeling, leading to symptomatic HF. These more recent studies have influenced concepts of the role of processes in thin, thick, and titin filaments in controlling tension development with each beat of the heart. Results of one line of investigations have altered perceptions of force generation by stressing a more novel role for the thick filament structural state and signaling than previously appreciated [24,25]. Employing largely biochemical approaches and in vitro methods, these studies with healthy rabbit heart tissue identified and characterized the existence of a population of cross-bridges in an interacting head motif (IHM) promoting a super-relaxed state (SRX) with low ATPase activity and sequestered from participating in force generation [24]. These SRX myosin heads are believed to exist together with a population of myosins in a disordered relaxed state (DRX) available for interaction with thin filament sites upon activation by Ca^2+^ binding to cardiac troponin C (cTnC). Variations in the thick filament DRX/SRX populations have been theorized to be responsible for adjustments in the inotropic state in physiological conditions such as adrenergic stimulation, and for altered tension development in HF, HCM, and DCM [26,27,28,29,30]. Shifts in the SRX/DRX population have also been characterized as being responsible for inotropic effects of myosin activators and inhibitors [26,28,29].

In contrast, earlier and more recent studies employing a high-resolution nano-meter X-ray analysis have emphasized a transition of myosins from an OFF to an ON state by mechano-sensing with a minimal role for the IHM theory in living skeletal and cardiac muscle preparations [31]. Evidence from the X-ray data supported the conclusion that in relaxation, myosins are in an OFF state with heads in helical order, folded close to the thick filament surface [32,33]. A few myosins were proposed to be scanning for thin filament sites. Mechanical strain on the thick filament induced by the initial binding of force-generating cross-bridges with the onset of thin filament activation in the heartbeat was theorized to activate the transition of the OFF myosins to a force-generating ON state. Additional evidence relating to mechano-sensing of the thick filament has been reported by Caremani et al. [34], who demonstrated that inotropic interventions phosphorylating cardiac troponin I (cTnI) and cardiac myosin binding protein-C (MyBP-C3) had no effect on the state of myosin in relaxed muscle. These findings do not support a role for MyBP-C3 phosphorylation previously suggested to modulate and promote the transition from SRX to DRX states [27]. Moreover, Caremani et al. [34] stress that the lack of effect of MyBP-C3 phosphorylation on the thick filament state in diastole indicates that the regulatory mechanism is downstream of thin filament activation and fits with a model in which variations in thick filament strain are significant determinants of force generation. Thus, the gain in the interaction energies induced by the strain-dependent activation of the thick filament is thought to be a modulated variable, which can be affected by sarcomere length, post-translational modifications (PTMs) in thin and thick filaments, and shifts in sarcomere protein isoforms associated with altered physiological and pathological remodeling. As discussed below, recent X-ray diffraction investigations testing the effect of OM in porcine trabeculae support the prominent significance of the OFF/ON versus the SRX/DRX state [35]. These studies demonstrated a transition of the resting cross-bridges from OFF to ON states induced by OM, but there was no evidence that this transition involved a modification in SRX/DRX states. Moreover, the authors emphasized that the OFF state and SRX state are not equivalent. These findings indicate the need for a more thorough consideration of the mechanisms of the activation of thick filament function considering the possibility of different conclusions depending on the methods of approach. The interpretation of the detailed structural information derived from cryo-electron tomography of relaxed native cardiac sarcomeres provides a cogent example of the need for caution in the interpretation of data related to thick filament structure/function relations [36]. Whereas the resolution and imaging are impressive and advance understanding of the three-dimensional structure of myosin, titin, and MyBP-C3, the studies were carried out in isolated myofilaments in the presence of 50 µM mavacampten. The results showed no interaction between titin and MyBP-C3 that others have reported [37]. More recently, these studies have been repeated in thick filament preparations without mavacamten [38]. These studies without mavacamten identified three populations of motors in relaxed thick filaments indicated as equivalent to SRX, DRX, and active myosins [38]. In both these studies, the discussion of the data included the IHM concept but did not include the ON/OFF concept of thick filament activation derived from X-ray data in preparations with myofilaments functioning in an intact cellular environment. Other examples are recent publications on the mechanism of the Anrep effect and the Frank–Starling relation, which reviewed the literature, concluding that there is a major role of the SRX/DRX transition responding to load with no reference to the ON/OFF mechanisms [39,40].

## 3. Cross-Bridge ON/OFF States and Control of Tension/Wall Stress

**Figure 1:**
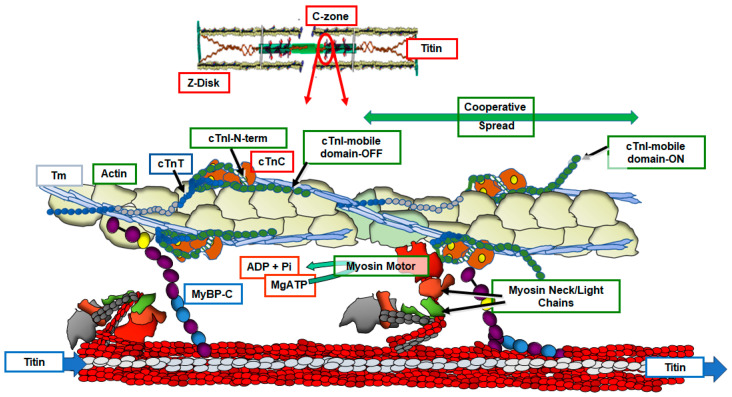
Near-neighbor C-zone cardiac regulatory units in OFF and ON states. The left regulatory unit is in an end-diastolic state (relaxing levels of cellular Ca^2+^) with low levels of the phosphorylation of key control elements in cTnI, MyBP-C3, and titin. See the text and the legend and diagram in Figure 2 for further discussion. Relaxed myosin heads are folded close to the thick filament in an OFF state with force-generating reactions with actin impeded by Tm. This Tm steric block is by the cTn complex extending along the functional unit with multiple inhibitory interactions involving N- and C-terminal regions of cTnI and cTnT interacting with cTnC, actin, and Tm. Head-to-tail overlap of contiguous Tms interacts with regions of the cTnT N-terminus reducing the possibility of cooperative activation along the sarcomere strand. The low-Ca^2+^ blocked state of the thin filament is tempered by interactions of N-terminal domains of MyBP-C3 with actin–Tm that partially reduce the steric block. This interaction is reduced with elevated Ca^2+^ and abolished with the phosphorylation of MyBP-C3. Not shown is the possibility for direct interactions of MyBP-C3 with cTnI, proposed to affect coordinated effects of the phosphorylation of these proteins. The stretch of titin elastic domains induces resting tension and diastolic pressure; titin C-zone interactions with MyBP-C3 and myosin potentially modulate the OFF state. There are also interactions of MyBP-C3 domains with the regulatory light chain (RLC) of myosin in a phosphorylation-dependent manner. Complex allosteric, steric, and cooperative mechano-sensing mechanisms involving all the major sarcomere proteins activate sarcomere force and shortening. Acting allosterically, elevations in Ca^2+^ binding to cTnC trigger the modification of protein–protein interactions releasing a nearly complete Tm steric block and inducing the reaction of force-generating cross-bridges with the thin filament. According to the ON/OFF theory of tension control, these initial cross-bridge reactions induce a mechanical strain on the thick filament promoting more cross-bridges to engage and develop tension and further activate the thin filament. The activation of a near-neighbor functional unit occurs by a cooperative spread along the strands of sarcomeres aided by effects’ cross-bridge-dependent thin filament activation mechanisms and by Tm-Tm interactions between contiguous sarcomeres. The reactions responsible for the level of the relaxed state and the intensity of the active state are variables controlled by the gain in mechano-sensing by load and length and by neuro-humoral-induced post-translational mechanisms. These homoeostatic mechanisms are exquisitely sensitive to mutations triggering DCM.

**Figure 2:**
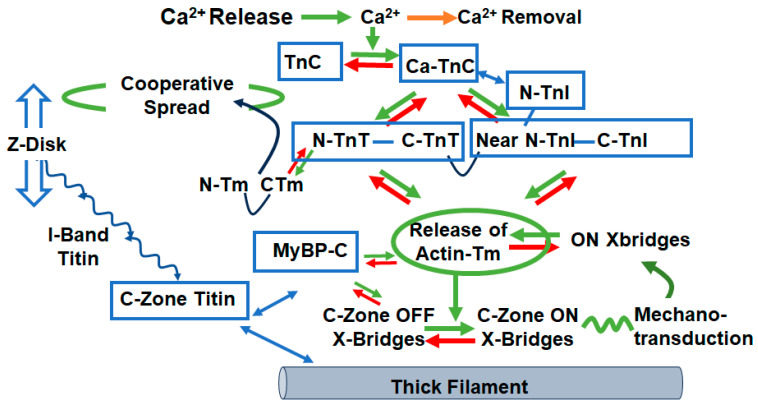
Sarcomere protein–protein interactions in the transition between diastole and systole. The forward (green arrows) and reverse (red arrows) flow of reactions according to the ON/OFF theory of tension generation is initially triggered by Ca^2+^ binding to cTnC with the generation of tension by a few cross-bridges promoting mechano-transduction. The increased strain in the thick filament leads to more force-generating cross-bridges and establishing tension during the heartbeat. The modulation of this process is pictured to occur downstream of these interactions that alter the gain in reactions. This variation in gain occurs with alterations in protein–protein interactions in the biochemical environment, changes in sarcomere length, signaling via post-translational mechanisms, and the expression of natural and DCM-linked isoforms. Small molecules that activate tension are also thought to modify this gain. See text and Figure 1 for further discussion and information.

Implications of the ON/OFF mechanism in various states of cardiac function indicate the need for a reconsideration of previous reviews of how the integrated interactions among sarcomere proteins control gradations in cardiac wall stress/tension and contraction/relaxation dynamics. There is general agreement that the control of tension in striated muscle in both relaxation and the activation of tension with sliding of myofilaments requires mechanisms involving all three major myofilaments, thin, thick, and titin. However, there is not a clear consensus on the fundamental mechanisms. As summarized above, we focus here on compelling evidence provided by the X-ray data highlighting the dominance of the ON/OFF mechanisms in contrast to shifts in the SRX/DRX ratio [34,35]. Integrated interactions among sarcomere proteins’ controlling function are illustrated in Figure 1 and the reaction flow in Figure 2, based on high-resolution structure [41,42] and recent reviews [43,44]. Figure 1 illustrates a snapshot of the sarcomeres of the C-zone where MyBP-C3 is localized in an end-diastolic state (relaxing levels of cellular Ca^2+^) at a basal heart rate with low levels of the phosphorylation of key control elements in cTnI, MyBP-C3, and titin. The diagram in Figure 2 is a companion to Figure 1, which depicts these protein–protein interactions as reactions promoting tension and inducing relaxation. In Figure 1, myosin heads are shown as uniformly folded close to the thick filament proper. With regulatory sites of cTnC free of bound Ca^2+^, functional regulatory units localized in the two strands of the thin filament and made up of a 7:1:1 ratio of actins: the tropomyosin (Tm) and troponin (Tn) complex established a thin-filament-inhibited or so-called B-state with Tm in a configuration sterically blocking the reaction of myosin with actin [45]. We note that the regulatory units in each strand are not in register and not equivalent [42,46]. As shown, the cTn complex extends along the entire functional unit with each end of the complex having functional roles ensuring a thin filament off state. The C-terminal domains of cTnI contain a highly basic inhibitory peptide (Ip) and a switch peptide (SwP) extending from the cTn core domain and interacting with actin/Tm holding Tm in the B-state. Near N-terminal regions of cTnI extending along the cardiac troponin T (cTnT) C-terminal region (I-T arm) position cTnT in a configuration also promoting the binding and immobilization of Tm. There are also interactions of regions of the cTnT N-terminus with the head-to-tail overlap of contiguous Tms forming a key element in signaling of the diastolic state and reducing the possibility of cooperative activation along the sarcomere strand [47,48]. In diastole with low levels of intracellular Ca^2+^, there is evidence of the modulation of this inhibited state by the interactions of the N-terminal domains of MyBP-C3 with actin–Tm that move Tm away from the blocking configuration [46,49,50]. This interaction is weakened in high Ca^2+^ and abolished with the phosphorylation of MyBP-C3 [51]. It has also been proposed that there are direct interactions between MyBP-C3 and cTnI that coordinate effects of the phosphorylation of these proteins [52]. However, it is not clear how these interactions may modulate inhibition. The role of titin must be considered in the establishment of the diastolic state. At end diastole, sarcomere length is relatively long, and the stretch of titin elastic domains induces resting tension and diastolic pressure [53]. Interactions of titin with C-terminal domains of MyBP-C3 myosin rods may modulate thick filament packing. Specific interactions of three C-terminal domains with specific super-repeats on titin establish the localization of cMyBPC in its striped configuration in the C-zone of the sarcomere [37]. There are also interactions of MyBP-C3 domains with the regulatory light chain (RLC) of myosin in a phosphorylation-dependent manner [54]. The stability of the relaxed OFF state of the thick filament may involve these interactions of the RLC, titin, and MyBP-C3 that modulate head–head interactions as well as tail–tail interactions that deploy myosins in the relaxed state folded close to the thick filament [55].

The transfer from the diastolic to the systolic state involves complex allosteric, steric, and cooperative mechano-sensing mechanisms involving all the major sarcomere proteins in various roles. This ensures a rich array of mechanisms for beat-to-beat tuning of dynamics to HR and the modulation of the coupling of cardiac function to the metabolic needs of the cells of the body. Membrane excitation and elevations in intracellular Ca^2+^ promote allosteric interactions in both thin and thick filaments signaling the potential interactions of cross-bridges with actin triggered by a Ca^2+^-dependent release of the steric block imposed by Tm. With Ca^2+^ localized in its regulatory hydrophobic pocket, cTnC enters a rotation that aids binding of the Sw peptide and the translocation of the Ip away from sites of inhibition. Recent cryo-electron microscopy (cryo-EM) evidence also indicates a structural interaction between the cTnC N-terminus and the cTnI-C-terminus [46]. In turn, the mobile cTnI C-terminal domain moves away from a position no longer tethering Tm in a blocking position. The relief of inhibition includes a reconfiguration of the complex that alters interactions of C-terminal regions of cTnT with cTnI, cTnC, and Tm that alters interactions of N-terminal regions of cTnT at the overlap of N- and C-terminal Tm regions. These interactions induce a landscape of protein–protein interactions promoting a cooperative spread of activation along the strand of sarcomeres. However, despite the significant release from their inhibited state, thin filament activation is not complete as full activation requires binding of force-generating cross-bridges [46,56]. A fundamental tenant of the ON/OFF mechanism of the promotion of tension development is that the initial activation of some cross-bridge interactions produces a mechanical strain inducing a transition of other cross-bridges into the force-generating cycle. The efficacy of this strain-dependent activation is stated to be dependent on the gain of the protein–protein interactions [34]. This “gain” with variations in the energies of interaction among the thick filament components provides a regulatory device critical to control by the sarcomere load and length, PTMs, and biochemical environment. Imagining the arrows in Figure 2 to be of variable intensity, the graphic provides an illustrative example of the breadth of possible regulatory mechanisms available to maintain a homeostatic range of cardiac function under varying hemodynamic loads. Evidence that the peak tension in a twitch of intact trabeculae involves only ~10% of the available myosin motors in the sarcomere C-zone indicates the extent of the reserve in the contractile state [57].

## 4. Implications of the ON/OFF Theory in Modulation of Tension and Dynamics by Dominant Myocardial Regulatory Mechanisms

Unraveling the variations in the gain in the detailed mechanisms of the generation and control of tension/wall stress in the heart has wide ranging impact on the understanding of physiological and pathological processes in the heart as well as diagnoses, patient stratification, and the application of personalized medicine. An extensive consideration of this impact is beyond the scope of this review/commentary. We choose to focus on the impact of the ON/OFF theory of tension generation in familial DCM emphasizing the modulation of function by length and load on the sarcomeres, and by the protein phosphorylation of major sarcomere proteins. Our rationale is also that these mechanisms underpin the search for sarcomere-related therapies in the clinical course of DCM. Figure 1 and Figure 2 illustrate the unfolding of the activation process in the ON/OFF mechanism in the context of a basal physiological environmental and mechanical state with a low influence of PTMs. Common sarcomere-related control mechanisms that modulate this basal state include changes in sarcomere length (Frank–Starling mechanism) and neuro-humoral adrenergic stimulation. Thus, it is important to discuss how the consideration of signaling in the ON/OFF state transitions alters the understanding of these control mechanisms, whether and how they are modified in DCM, and whether and how they are altered by sarcomere activators.

### 4.1. Regulation by Sarcomere Length and Load

The understanding of mechanisms of modifications in the sarcomere response to Ca^2+^ as a key element in the Frank–Starling relation is not complete. Yet, there is general agreement that the stretch of titin instigates the increase in developed pressure with increased ventricular filling. Possible mechanisms of the effect of titin stretch in diastole include the modulation of thin filament and thick filament proteins including MyBP-C3. As indicated in Figure 1, C-zone regions of titin interact with myosin heavy and light chains and MyBP-C3. There is also the interaction of the Z-repeats of titin and with the Z-disk proteins telethonin, alpha-actinin, and actin [22]. Evidence indicates that these interactions sense the compliance of titin and induce signaling, resulting in an alteration of the number of force-generating cross-bridges. Moreover, DCM associated with truncating mutations in titin has been theorized to be significantly related to altered length-dependent activation (LDA) and modified by titin phosphorylation [21]. Early results of studies of effects of titin strain on LDA concluded an important role for a stretch-induced alteration in inter-filament spacing [58]. The idea was that increased compliance of titin blunted an effect to move myosin heads nearer to the thin filament. The phosphorylation of MyBP-C3 was also proposed to modify LDA by a mechanism shown by low-angle X-ray studies of skinned trabeculae to move myosin heads closer to thin filaments [59,60]. However, more recent investigations of intact muscle preparations have not supported this mechanism. From results of the X-ray investigation on the effects of changes in sarcomere length, Caremani et al. [34] and Brunello et al. [57] demonstrated no effects of a sarcomere length change in the diastolic OFF state just before the contraction or isoproterenol treatment of myosins in relaxed preparations despite a robust effect of both interventions on tension development. They note that with preparations treated with isoproterenol, there was an effect on meridional reflections attributed to MyBP-C3 and troponin. An X-ray analysis reported by Ait-Mou et al. [61] also did not demonstrate changes in inter-filament spacing or a movement of myosin heads toward the thin filament with stretching of electrically stimulated mouse heart muscles. However, other reflections indicated significant structural rearrangement in myosin heads not found by others [34,57]. Other lines of evidence point to a mechanism in which titin strain signals the thin filament complex to promote an increase in tension with an increase in sarcomere length. Evidence for this signaling comes from the data demonstrating that the troponin complex senses length not only by structural rearrangements but also by enhanced cTnC Ca^2+^ binding. Employing RBM20-deletion mice that express the N2BA highly compliant titin isoform, Li et al. [62] reported that stretching induced a diminished FRET signal at the cTnI-cTnC interface compared to controls with relatively higher passive tension. The signal was also induced by strong cross-bridge binding. Taken together, the data summarized above indicate that a change in sarcomere length signals the thin filament to increase the population of force-generating cross-bridges and potentially increase the gain in mechano-sensing affecting thick filament strain. This mechanism supports the ON/OFF theory in that length-dependent activation occurs downstream of thin filament signaling and depends on the compliance of titin. A role for the thin filaments and MyBP-C3 in length-dependent sarcomere signaling in the Frank–Starling relation is supported by data reporting alterations in length-dependent force development independent of a change in titin compliance within myofilaments controlled by a deletion mutant of cTnT (cTnT-ΔK210), by the mutant cTnT-R171W, and by a mutation in cTnC (cTnC-G159D) [63,64,65]. All these mutations are linked to DCM. However, the cTnT mutations decrease the myofilament response to Ca^2+^, whereas the mutation in cTnC induces an increase in Ca^2+^ sensitivity [65].

In the context of load and length sensing and signaling, it is important to mention the significant effects of DCM-linked variants of *DES*, which expresses the intermediate filament desmin. Reviews by Pyle and Solaro [66] and Brodehl et al. [7] summarize the desmin cytoskeletal network, which provides extensive scaffolding among myocytes, between myocytes and the extracellular matrix, and among the sarcolemma, mitochondria, Z-disks, and nuclear envelope. Disorders in the desmin network promote maladaptive signaling, remodeling, and growth in DCM together with associated defects in sarcomere assembly and the induction of apoptosis. A common histological abnormality is the formation of desmin aggregates, likely to interfere with homeostasis of multiple functions in myocytes. As emphasized by Brodehl et al., the complexity and extent of the pathological mechanisms produces a formidable challenge in developing therapeutic interventions.

### 4.2. Regulation by Sarcomere Protein Phosphorylation

Among the many sites and types of PTMs in sarcomere proteins, phosphorylations of cTnI and MyBP-C3 at their unique N-terminal domains have taken on prominent roles in tuning tension and dynamics to common physiological demands on the heart. Both mechanisms have been well studied and discussed for some time but have been challenged by recent data [34,52]. In the case of MyBP-C3, we mentioned above that a predicted shift in the SRX/DRX ratio with phosphorylation was not detected in the intact electrically stimulated trabeculae studied by nano-meter X-ray diffraction [34]. The results of the X-ray analysis came to a different conclusion than previous studies with detergent-extracted muscle preparations. These studies concluded that in relaxed sarcomeres, the phosphorylation of MYBP-C3 releases it from binding to the thin filament, inducing its binding to the S-2 region of myosin, thereby promoting the population of myosin motors in the DRX state [67]. In the ON/OFF mechanism, there is a shift in myosin heads with the adrenergic stimulation and phosphorylation of MYBP-C3, but not in relaxation. Thus, thin filament activation with an increase in thick filament strain precedes a downstream shift in MyBP-C3 binding to myosin. Although Caremani et al. [34] noted no modifications of the OFF state with adrenergic stimulation, there were altered reflections of both MyBP-C3 and cTnI in these experiments.

Apart from these findings in the X-ray analysis, the detailed mechanisms and effects of cTnI phosphorylation at its N-terminus Ser residues have also been challenged in study [52]. For nearly 50 years, although there have been reports of differences in the altered molecular signaling with cTnI phosphorylation at S23 and S24, there has been general agreement that the phosphorylation is a negative regulator of the myofilament response to Ca^2+^ and an important element in the lusitropic response to β-adrenergic stimulation [68,69]. Results of studies in the natural cellular environment in preparations with and without the expression of phospholamban demonstrated that β-adrenergic stimulation enhanced relaxation in mechanisms associated with the desensitization myofilament response to Ca^2+^ [70,71]. Current theories are driven by the dominant idea that cTnI phosphorylation induces lusitropy in cardiac dynamics important in tuning cardiac dynamics to HR and offsetting effects of HCM and DCM [69,72,73]. An example of the proposed molecular mechanism was reported by Pavadai et al. [74] in a simulation of protein–protein interactions employing molecular dynamics and molecular docking. They concluded that at sub-maximum myocyte Ca^2+^ concentrations, unphosphorylated cTnI is highly mobile. With the pseudo-phosphorylation of the N-terminal domain at Ser 23 and Ser 24, they predict that this unique region of cTnI interacts with both the N-lobe of cTnC interfering with Sw peptide binding and with Tm, resulting in the promotion of the off state of the thin filament. In stark contrast to these predictions, studies by Sevrieva et al. [52] employing isolated myofibrils and skinned trabeculae preparations with authentically phosphorylated cTnI and MyBP-C3 reported that cTnI phosphorylation alone increased Ca^2+^ sensitivity of trabeculae force generation at short and long sarcomere lengths. When both cTnI and MyBP-C3 were phosphorylated, compared to controls, there was an increase in force only at long sarcomere lengths. Moreover, other in vitro studies reported a direct phosphorylation-dependent interaction between the N-terminal domains of MyBP-C3 and cTnI. In their paper, Sevrieva et al. [52] indicate that pseudo-phosphorylation does not faithfully reproduce the effect of authentic phosphorylation without a direct test of this assertion in cTnI. In addition, there was no mention of the criticism of skinned fiber preparations by Caremani et al. [34] in relation to mechanisms of effects of MyBP-C3 phosphorylation. It is important to clarify whether cTnI phosphorylation activates or inhibits sarcomere force development since a series of publications demonstrated an uncoupling between effects of sarcomere protein phosphorylation and cardiac mechanics in the promotion of DCM progression [73]. There are several issues regarding the conclusion reported by Sevrieva et al. [52], which indicate a need for a re-evaluation of their conclusions. A major issue is the lack of evidence for their findings in the integrated in situ regulation of the heartbeat. In Figure S2, the authors reported a relatively low efficiency of exchange (35–50%) of bis-phosphorylated cTnI, and the native cTnI appears to be highly phosphorylated (Figure 1A in the paper). This suggests that they might be assessing the effect of only a small additional increase in cTnI phosphorylation. Additionally, the isolated trabeculae did not demonstrate RLC phosphorylation (Figure S6 in their manuscript), which is not explained. This lack of RLC phosphorylation may impact the results and conclusions from the fibers exchanged with bis-phosphorylated TnI. Earlier work reported by Sevrieva et al. [75] concluded a role of MLCK in complex mechanisms affecting the relative effects of cTnI and RLC phosphorylation. Moreover, fully dephosphorylated myofilament preparations were treated with PKA but there was no thorough analysis of sites of phosphorylation that are apparent in high molecular regions of the gels. Finally, some degradation of cTnI was noted in Figure 2C of their manuscript and this may be a cause for concern.

## 5. Research Challenges in the Quest to Develop Sarcomere-Targeted Drugs Treating DCM

Landim-Viera and Knollman [76] made the following statement in a recent editorial: “The search for small molecules that increase contractile function by directly targeting the muscle of the failing heart can be considered the holy grail in drug development”. Research challenges in the search for agents effective in DCM therapy include the lack of a unified theory of the control of tension development, the evaluation of the role of TTI as a predictor of DCM, and differing views of effects of myofilament protein phosphorylation. Challenges include some data that mechanistic conclusions from studies with detergent-extracted fibers and fiber bundles may not be obtained in the intact myocardium. There are criticisms of commonly employed approaches including the pseudo-phosphorylation and substitution of Ala residues for Ser/Thr phosphorylation [52]. We discuss these research challenges related to the understanding of disease mechanisms and the quest for development of sarcomere-targeted treatments in DCM. We have limited the discussion to agents targeting thin filaments and thick filaments including MyBP-C3.

### 5.1. Targeting Thin Filaments in DCM Therapy

Although many recent papers continue to refer to the “modulation of sarcomere contractility” with small molecules acting at sarcomere proteins as a new or recent therapeutic opportunity [77,78,79,80], research in this area began more than 40 years ago. Solaro and Ruegg introduced a research direction in the development of inotropic agents with the hypothesis that small molecules can enter the intracellular space and bind directly to myofilament regulatory proteins inducing an increase in the sarcomere response to Ca^2+^ [81]. Experiments reported by Solaro et al. [82] supported this hypothesis with evidence for the existence of a cTnC domain acting as a receptor for the thiadiazinone EMD57033. They demonstrated the enantiomeric separation of the effects of the (+/−) EMD57033 with only the (+) enantiomer active in increasing the myocyte contractility, myofilament response to Ca^2+^, cTnC Ca^2+^ binding, and thin filament sliding in the motility assay [82]. These data provided a proof of principle for targeting sarcomere proteins in HF [82]. Solution nuclear magnetic resonance (NMR) studies of stereospecific interactions correlated the enantiomeric separation by demonstrating functionally significant key interactions of only the EMD (+) chiral group located deep in a hydrophobic pocket of cTnC [83]. These agents were originally termed “Ca^2+^ sensitizers” and more recently “sarcomere activators” [84,85]. The proposed advantages of this approach included an increase in tension with little or no change in the Ca^2+^ transient. This action was described as an energy-sparing effect with an increase in tension with no change in oxygen consumption related to the reduction in the load on the sarcotubular Ca^2+^ pump. Moreover, unlike conventional inotropes that increase intracellular Ca^2+^, the sensitizers were expected to be non-arrhythmogenic. However, this idea has been challenged in studies reporting that elevated myofilament Ca^2+^ sensitivity may indeed be arrhythmogenic [86]. Examples of agents targeted to cTnC that progressed successfully through clinical trials are levosimendan (Simdax) developed by Orion Pharma/Abbott Labs [87,88] and pimobendan (Acardi; Vetmedin) developed by Boehringer Ingelheim/Nippon and manufactured by Boehringer Ingelheim, Athens, GA USA [89]. An unexpected off-target effect in the pharmacological profile of these Ca^2+^-sensitizing compounds was the inhibition of phospho- diesterase III (PDE III) and elevations in cellular cAMP levels [89,90,91,92]. This pleiotropic property led to the designation of pimobendan and levosimendan as inodilators [93,94]. However, with PDE III inhibition, there are elevations in HR, which has the concern of being arrhythmogenic, especially in compromised cardiac function [11]. In view of this concern, continued efforts to develop sarcomere activators ruled out agents with PDE III inhibition. Reports in the literature have generated mixed results on the efficacy of pimobendan and levosimendan in DCM. The inodilator pimobendan was reported to be of benefit in the cTnT-ΔK210 DCM mouse model despite the lack of evidence that there was an increase in the myofilament Ca^2+^ response [95,96]. In a recent study employing this model [95], there was a prevention of cardiac remodeling in compensated HF with a significant increase in the life span regardless of the stage of treatment. However, at the end stage, there was an increase in sudden death. In contrast, treatment with pimobendan (marketed as Vetmedin^®^ Boehringer Ingelheim, Athens, GA, USA) is a preferred and effective therapy for dogs with DCM of various etiologies [97]. Whatever the case, with the lack of a consensus on the effects of the cAMP-dependent phosphorylation of MyBP-C3 and cTnI, the risk/benefit of the inodilators is not clear.

### 5.2. Targeting the Troponin Complex Avoiding PDE III Inhibition

The quest to find a troponin activator without the negative effects of PDEIII inhibition continues with early successes in moving forward in the development of small molecules presumably acting at the cTnI-cTnC interface. Improvement in the DCM phenotype in mice expressing variants of cTnC and cTnI that increase the myofilament response to Ca^2+^ provided a proof of principle that this approach may be successful. We have employed the genetic modification of thin filament regulation to inform potential approaches to DCM therapy in which there is an increase in the myofilament Ca^2+^ response by expressing slow skeletal TnI (ssTnI) to alter the cTnC-cTnI interaction [98]. Compared to cTnI–myofilaments, myofilaments controlled by ssTnI demonstrate an increased response to Ca^2+^ [69,99]. Double-TG mice expressing DCM-linked Tm-E54K and ssTnI had a significantly improved response to Ca^2+^ and improvements in the phenotype of the DCM model. Our studies modifying cardiac troponin isoform expression are related to screening for Tn activators employing a TnI-TnC chimera, thus probing the modulation of this interaction [100,101]. In a similar approach, Powers et al. [12] tested whether the expression of a modified cTnC (cTnC-L48Q), which enhances the myofilament Ca^2+^ response and increases tension, can improve maladaptive effects of the expression of a DCM-linked mutant Tm-D230N in a mouse model. Compared to controls, double-transgenic mice expressing both the mutant Tm and cTnC-L48Q showed significantly elevated TTI and improvements in the maladaptive effects of DCM.

Promising troponin-activating compounds have been developed by investigators at Cytokinetics using a high-throughput assay employing ATPase activity of cardiac myofibrils [102]. These investigators have reported detailed findings in the development of CK-963 and discuss in their recent paper next steps in future clinicals trial with follow-up compounds [102]. Studies reported by He et al. [79] provide an example of advances in development of small molecules acting as thin filament activators without PDE III inhibition. They reported that the activation of troponin with a small molecule called TA1 increased myofilament Ca^2+^ sensitivity, increased tension in intact myocardial preparations, and elevated developed pressure with a reduction in HR and left ventricular end-diastolic pressure. In contrast with the inotrope dobutamine that increased cAMP and was energy-wasting, TA1 had no effects on the phosphocreatine/ATP ratio, or ΔG_~ATP_. Another study developed a high-throughput assay probing effects of chemical libraries on the cTnI-cTnC interaction with fluorescent probes [103]. Results identified NS5806 as a compound that forms a complex with the N-lobe of cTnC promoting Sw peptide binding without affecting Ca^2+^ binding. It remains unclear whether these effects of NS5806 occur in situ. Another compound identified in NMR investigations as a troponin activator is RPI-194 [104]. Although studies with RPI-194 provide clues to approaches, its effects are not restricted to cardiac myofilaments.

### 5.3. Myosin Heavy Chain as a Drug Target

More recently, biotech and pharma have made significant progress in shifting emphasis to targeting the myosin heavy chain with small molecules as a therapy in HCM, acquired HF, and DCM [77,85,105] Investigators such as Cytokinetics, Inc led the way with development of OM, an agent activating sarcomere tension by complex mechanisms discussed below [85]. Although trials did not achieve end points for transition to the clinic, the evidence that OM may be an effective therapy in acquired HF has stimulated more research. This further effort resulted in the development of danicamtiv (MYK-491), an agent in current clinical trials proposed to have a similar profile as OM, but different pharmacokinetics [76,77]. Danicamtiv has been proposed to recruit myosin motors from the OFF state and increase myofilament Ca^2+^ sensitivity, but this needs to be re-evaluated with techniques such as high-resolution X-ray diffraction in intact muscle. Moreover, studies need to be carried out in a variety of DCM mouse models in addition to the one employed in the study expressing a cTnC-I61Q variant with reduced Ca^2+^ binding, but which is not linked to DCM gene variants. It is of interest that in the clinical trial, effectiveness of treatment with MYK-491 includes patients with DCM gene variants expressing variants of the myosin heavy chain as well as titin.

There are some issues and considerations about the potential use of myosin activators in DCM. An important question is whether emerging myosin activators such as OM and danicamtiv are effective in genetic DCM progression. Agents targeted to activate myosin cross-bridges have gone through clinical trials showing some beneficial effects in human HF, but the population of patients in the cohorts with genetic DCM is unclear [29]. An issue that needs to be considered is whether there are long-term effects reducing the progress of the clinical course. Although more evidence is required, there are data indicating that long-term effects of OM administration may have palliative effects [106]. Seven days following a single infusion of OM in healthy adult rats El Oumeiri et al. [106] compared cardiac function and gene expression in the hearts with controls. Hearts of OM-injected rats had a prolonged ejection time and shortened pre-ejection period, with no changes in the ejection fraction, cardiac output, heart rate, and stroke volume. The data indicated a possible beneficial long-term effect via anti-apoptotic and anti-oxidative stress signaling. However, there was an increase in fatty acid oxidation with potential for increased oxygen consumption, and an increase in the expression of angiotensin II receptors-1 (AT-1) and AT-2 with a slight change in the ratio of the two receptors. There were no significant changes in proteins regulating Ca^2+^ movements, except for an increased expression of the alpha 1c subunit of the voltage-dependent Ca^2+^ channel (Cacna1c). In view of a lack of effect of OM on intracellular Ca^2+^ transients, the functional significance of this change is not clear. Moreover, tests of effects of OM in living tissue slices from human patients with HF showed that although there was an increase in contractility and the duration of contraction time, relaxation was impaired [107]. The relaxation prolongation worsened with increased beating rates and was suggested to possibly promote arrhythmias. However, in vitro studies reported a low probability of OM being pro-arrhythmic [108]. There is also a consideration emphasized by Tang et al. [14] in relation to mutations in myosin linked to DCM. They reported that OM had a reduced effect to stimulate cross-bridge function in preparations containing the β-myosin heavy chain with a DCM-linked mutation, F764L. These investigators concluded that mutation-specific modifications in sarcomere activation by OM need to be considered.

There are other views of the precise mechanism of action of OM in promoting tension development that are relevant to its therapeutic application in altering effects of disease-causing sarcomere genes. Potential effects on thin filament function indicate that myosin activators may be effective in DCM with diminished tension not associated with shifts in the myosin SRX/DRX populations. We reported in vitro studies that demonstrated that OM increased the Ca^2+^ sensitivity of Tm-E54K myofilaments [109]. Although also reported by others, the effect of OM to increase sarcomere Ca^2+^ sensitivity and modify cooperative activation is not generally discussed in considerations of its mechanism of action [110,111]. Kieu et al. [112] reported that at physiological temperatures, there is a prominent effect of OM in increasing skinned fiber sub-maximal tensions in the force–Ca^2+^ relation with no effect on maximum tension. Kieu et al. [112] attributed this effect to a mechanism in which the prolonged duty cycle associated with OM enhances cooperative thin filament activation, which is maximized at physiological temperatures disallowing an effect at saturating Ca^2+^ concentrations. We have also tested the effects of OM on the function of cardiac myocytes derived from human inducible pluripotent stem cells (hiPSC-CMs) from patients expressing the DCM-linked cTnT-R173W mutation [113]. We found that OM improved function and promoted F-actin assembly and content in the hiPSC-CMs. OM also reversed a depression in cross-bridge entry into the force-generating state in preparations reconstituted with either wild-type cTnT or recombinant cTnT-R173W. Whether myosin activators are therapeutic in DCM remains an open question. However, studies of Tm variants (Tm-T237S) linked to DCM by Barrick et al. [114] reported a decrease in the Ca^2+^ sensitivity of the ATPase activity in reconstituted preparations compared to controls. Treatment of these preparations with OM increased Ca^2+^ sensitivity, indicating that OM may be a useful agent in DCM. Moreover, Lehman et al. [115] have suggested the potential efficacy of danicamtiv and OM in DCM based on their effects in HF studies. It will be of interest to determine early and long-term effects in the DCM progression of the administration of these more recent sarcomere activators.

A question relating to the therapeutic efficacy of OM and other myosin activators is whether their use not only increases tension in DCM but also reduces fibrosis [4,116]. This is an important issue as fibrosis has been known for some time to be a predictor of mortality and hospitalization in DCM [1,117,118]. In view of a lack of evidence that myosin activators reverse fibrosis in systolic HF, it has been argued that a fibroblast-specific therapeutic approach is necessary [4]. This makes sense as fibroblast proliferation occurs in the absence of cell death. However, the percent of patients with the most common manifestation of fibrosis in the ventricular mid-wall wall is less than half the patient population. A related hypothesis is that feedback from fibroblasts to myocytes promotes a loss of sarcomere tension in DCM. Support for this hypothesis was demonstrated by experiments showing that a specific inhibition of fibrosis by the expression of p38 MAP kinase in fibroblasts partially restores the loss of sarcomere tension in a DCM model [4]. This is not surprising as it is expected that mechano-signaling occurs not only within cells but from cell to cell. Based on these findings, the proposal has been made that a fibroblast-specific therapy should be an adjunct therapy in DCM. However, this therapeutic approach requires determination in patients of whether fibrosis is present or not. An editorial providing a balanced view of the role of fibrosis in genetic cardiomyopathies has been provided by Frangogiannis [119].

### 5.4. Cardiac Myosin Binding Protein C as a Drug Target

In view of the versatility of MyBP-C3 actions in controlling cardiac contraction and dynamics, it is not surprising that efforts are underway to modulate its function with small molecules. Initial studies employing high-throughput approaches based on the use of fluorescence lifetime measurements of the actin-MyBP-C3 interaction have been reported [80]. Early evidence provides an approach with the identification of lead compounds with specificity for cardiac N-terminal C0-C2 regions of MyBP-C3 with or without phosphorylation. The effect to modify in vitro function required high concentrations of the agents, indicating that much work remains. Alternative strategies for maintaining function in the presence of mutations of MyBP-C3 have been summarized by Schlossarek et al. [120]. These include modulators of the ubiquitin proteosome system to reduce the presence of poison peptides, gene replacement therapy, modified anti-sense oligonucleotides, and targeting of RNA processing.

## 6. Future Directions and Concluding Remarks

Diverse concepts of control of sarcomere tension discussed here indicate a need for the re-examination of the causes and progression of DCM linked to sarcomere mutations as well as therapeutic approaches. It is apparent that this re-examination requires emphasis on studies integrating the myocyte protein activities and interactions in living muscle preparations. Caution has been indicated about the use of the pseudo-phosphorylation and removal and replacement of Ser/Thr residues in assessing the mechanism. The use of pig hearts that is increasing in the literature should also be emphasized. Lessons from the use of high-resolution X-ray diffraction techniques demand that hypotheses based only on biochemical and structural studies with myofilaments and myofilament proteins isolated from the cellular environment need to be tested in functioning myocardium ideally in a basal and stimulated state with and without regulation by DCM mutant proteins and with and without treatment with candidate therapies. Advances in transcriptomic and proteomic approaches provide a snapshot of a particular state but information on stages of the DCM progression with and without therapeutic interventions to test for pleiotropic effects would be ideal. Although not described here owing to limitations, there is a need for the consideration of reciprocal interactions of the myocytes with the mosaic of changes within the micro-environment [121,122]. The diagnosis of severity of DCM has been made with non-invasive measurement of fibrosis, which has been validated with well-documented determinations of the presence of fibrosis [118]. Clinical determinations within the micro-environment are close to reality with the use of emerging high-resolution time-resolved approaches including four-dimensional flow cardiac magnetic resonance imaging (CMR), diffusion tensor imaging (DTI), and hybrid positron-emission tomography–MRI [123]. Moreover, clinical workflows for precision medicine in genetic cardiomyopathy remain a work in progress with some papers outlining the needs in the field in the general population [124] and in ethnic racial groups [125]. There are also the important issues in the broad spectrum of DCM including sex-related pathologies and lifestyles such as alcohol consumption [126,127,128].

## Data Availability

No new data were created or analyzed in this study. Data sharing is not applicable to this article.
